# Revisiting Schrödinger’s fourth-order, real-valued wave equation and the implication from the resulting energy levels

**DOI:** 10.1098/rsos.230793

**Published:** 2023-12-20

**Authors:** Nicos Makris

**Affiliations:** Department of Civil and Environmental Engineering, Southern Methodist University, Dallas, TX 75276, USA

**Keywords:** matter-waves, real wave equation, flexural stiffness, atomic spectral lines, quantum mechanics

## Abstract

In his seminal part IV, *Annalen der Physik* vol. 81, 1926 paper, Schrödinger has developed a clear understanding about the wave equation that produces the correct quadratic dispersion relation for matter-waves and he first presents a real-valued wave equation that is fourth-order in space and second-order in time. In the view of the mathematical difficulties associated with the eigenvalue analysis of a fourth-order, differential equation in association with the structure of the Hamilton–Jacobi equation, Schrödinger splits the fourth-order real operator into the product of two, second-order, conjugate complex operators and retains only one of the two complex operators to construct his iconic second-order, complex-valued wave equation. In this paper, we show that Schrödinger’s original fourth-order, real-valued wave equation is a stiffer equation that produces higher energy levels than his second-order, complex-valued wave equation that predicts with remarkable accuracy the energy levels observed in the atomic line spectra of the chemical elements. Accordingly, the fourth-order, real-valued wave equation is too stiff to predict the emitted energy levels from the electrons of the chemical elements; therefore, the paper concludes that quantum mechanics can only be described with the less stiff, second-order, complex-valued wave equation.

## 1. Introduction

During his effort to construct a matter-wave equation that satisfies the quadratic dispersion relation between the angular frequency *ω* and the wavenumber $k\;(\omega =\hbar /(2m)k^{2}\;\mathrm{with}\;\hbar =h/(2\pi )\;\mathrm{where}\;h=6.62607\times 10^{-34}\;m^{2}\;{\mathrm{kg}\;s}^{-1}=\text{Planck's constant})$ Schrödinger in his part IV, 1926 paper [1,2] reaches a real-valued, fourth-order in space and second-order in time differential equation:

1.1 $${\left(\frac{1}{m}\nabla^{2}-\frac{2}{\hbar^{2}}V(r)\right)}^{2}\psi (r,t)+\frac{4}{\hbar^{2}}\frac{\partial^{2}\psi (r,t)}{\partial t^{2}}=0,$$

where *m* is the mass of the elementary, non-relativistic particle and *V*(**r**) is its potential energy that is only a function of the position **r**. In his 1926 paper [1], Schrödinger explains in his own words: ‘equation (1.1) is thus evidently the uniform and general wave equation for the field scalar *ψ*’. He further recognizes that his fourth-order equation (1.1) resembles the fourth-order equations of motion that emerge from the theory of elasticity and references the governing equation of a vibrating plate. More precisely, because of the three-dimensional geometry of atoms, the description of an electron orbiting the nucleus with equation (1.1) resembles the equation of motion of a vibrating shell [3–6] which had not been developed at that time.

For standing waves, the spatial and temporal dependence of the matter-wave can be separated:

1.2 $$\psi (r,t)=\psi (r)\;e^{\pm (i/\hbar )Et},$$

so that

1.3 $$\frac{\partial \psi (r,t)}{\partial t}=\pm \frac{i}{\hbar }E\psi (r,t);\;\frac{\partial^{2}\psi (r,t)}{\partial t^{2}}=-\frac{E^{2}}{\hbar^{2}}\psi (r,t).$$

In the interest of simplifying the calculations in the eigenvalue analysis of equation (1.1), in association that *V*(**r**) does not contain the time, Schrödinger [1,2] substitutes the second of equation (1.3) into equation (1.1) and recasts it in a factored form:

1.4 $$\left(\frac{1}{m}\nabla^{2}-\frac{2}{\hbar^{2}}V(r)+\frac{2}{\hbar^{2}}E\right)\left(\frac{1}{m}\nabla^{2}-\frac{2}{\hbar^{2}}V(r)-\frac{2}{\hbar^{2}}E\right)\psi (r)=0.$$

He recognizes that equation (1.4) does not vanish by merely setting one of the factors equal to zero given that each factor is an operator. Inspired by the factorized form of his original fourth-order wave equation (1.1) given by equation (1.4) in association with the structure of the Hamilton–Jacobi equation [7–12], Schrödinger reverts to the first of equation (1.3) to separate the time dependence and settles with his iconic second-order in space and first-order in time complex-valued wave equation [1,2]:

1.5 $$i\hbar \frac{\partial \psi (r,t)}{\partial t}=-\frac{\hbar^{2}}{2m}\nabla^{2}\psi (r,t)+V(r)\psi (r,t).$$

At the end of section §1 of his part IV, 1926 paper [1,2] Schrödinger indicates that for ‘a conservative system, equation (1.5) is essentially equivalent to equation (1.1), as the real operator may be split up into the product of the two conjugate complex operators if V does not contain the time’.

The above equivalence statement advanced by Schrödinger is not true, since the fourth-order, real-valued wave equation (1.1) is a ‘stiffer’ equation than the second-order, complex-valued equation (1.5), yielding higher eigenvalues and therefore higher energy levels.

The higher energy levels predicted by the stiffer fourth-order, real-valued wave equation (1.1) than those predicted by the classical second-order, complex-valued Schrödinger equation (1.5) are shown in this paper by computing the energy levels of a one-dimensional elementary particle, *ψ*(*x*, *t*), trapped in a square well with finite potential *V*. The paper shows that the one-dimensional version of Schrödinger’s original fourth-order, real-valued equation is equivalent to the governing equation of a vibrating flexural-shear beam [13,14]. By splitting the fourth-order, real-valued operator into the product of two conjugate second-order, complex-valued operators and upon retaining only one of the complex operators, Schrödinger [1,2] essentially removed from his original fourth-order equation (1.1) its ‘flexural stiffness’ and left it only with ‘shear stiffness’.

In the view of the many predictions with remarkable accuracy of Schrödinger’s second-order, complex-valued equation (1.5) for the atomic orbitals of the chemical elements and other features of the Periodic Table [15–19] in association with the higher energy levels predicted from his original fourth-order, real-valued equation (1.1) (therefore, apparently incorrect), this paper offers a straightforward explanation why quantum mechanics can only be described with complex-valued functions—a finding that is in agreement with more elaborate recent studies that hinge upon symmetry conditions of real number pairs [20], the de Sitter algebra [21] or involve entangled qubits [22–24].

This paper shows in a simple, straightforward manner that Schrödinger’s original fourth-order, real-valued wave equation (1.1), which is the simplest possible real-valued wave equation that satisfies the quadratic dispersion relation $\omega =\hbar /(2m)k^{2}$, is too stiff to predict the energy levels that correspond to the observed atomic line spectra (infrared, visible and ultraviolet) of the chemical elements. By splitting the fourth-order, real-valued operator of equation (1.1) into the product of two conjugate second-order, complex-valued operators, Schrödinger [1,2] extracts a more flexible equation than his original fourth-order, real-valued equation (1.1) at the expense of being complex-valued—that is, his iconic equation (1.5) which predicted correctly the energy levels of the hydrogen atom; and subsequently made a wealth of fundamental predictions as manifested by the features of the Periodic Table of the chemical elements [15–19,25].

The question that deserves an answer is how Schrödinger developed the remarkable intuition to proceed from the onset of his efforts with a complex-valued equation for matter-waves—that is, only the one factor of the split fourth-order, real-valued equation; which while complex-valued, is flexible enough to predict the correct frequencies manifested in the observed atomic line spectra of the chemical elements in the years to come and abandoned his original fourth-order, real-valued equation that its predictions were apparently never explored.

## 2. The ‘flexural-shear beam’ equation for matter-waves

In the interest of illustrating that the fourth-order, real-valued wave equation (1.1) is a stiffer equation than Schrödinger’s second-order, complex-valued equation (1.5), we consider for simplicity a single elementary, non-relativistic particle with mass *m* > 0 in one dimension moving along the positive direction, *x*, within an energy potential *V*(*x*). The total energy of the elementary particle, *E*, is described with its Hamiltonian,

2.1 $$E=H(x,p)=\frac{\;p^{2}}{2m}+V(x),$$

where *p* = *m* d*x*/d*t* is the momentum of the elementary particle and *p*^2^/(2*m*) = (1/2)*m*(d*x*/d*t*)^2^ represents its kinetic energy. Using Einstein’s [26] quantized energy expression, E=hν=ℏω, and de Broglie’s [27] momentum–wavelength relation, p=h/λ=ℏk, where *k* = 2*π*/*λ* is the wavenumber, the Hamiltonian of the elementary particle given by equation (2.1) in the absence of a potential (*V*(*x*) = 0) yields

2.2 $$\omega =\frac{\hbar }{2m}k^{2}.$$

Equation (2.2) leads to a quadratic dispersion relation for matter-waves as opposed to the linear dissipation relation, *ω* = *Ck*, of electromagnetic waves of shear waves in a solid continuum.

The simplest expression for a matter-wave travelling along the positive *x*-direction is *ψ*(*x*, *t*) = *ψ*_0_ e^i(*kx*−*ωt*)^ and upon using that k=p/ℏ and ω=E/ℏ,

2.3 $$\psi (x,t)=\psi_{0}\;e^{\left(i/\hbar \;)(px-Et\right)}.\;$$

The time derivative of equation (2.3) gives

2.4 $$\frac{\partial \psi (x,t)}{\partial t}=-\frac{i}{\hbar }E\psi (x,t).$$

Substitution of the expression for the energy, *E*, given by equation (2.1) into equation (2.4) gives

2.5 $$i\hbar \frac{\partial \psi (x,t)}{\partial t}=\left(\frac{\;p^{2}}{2m}+V(x)\right)\psi (x,t).$$

The second space derivative of equation (2.3) gives

2.6 $$\frac{\partial^{2}\psi (x,t)}{\partial x^{2}}=-\frac{1}{\hbar^{2}}p^{2}\psi (x,t),$$

and substitution of the quantity *p*^2^*ψ*(*x*, *t*) from equation (2.6) into equation (2.5) yields the one-dimensional version of the time-dependent Schrödinger equation given by equation (1.5):

2.7 $$i\hbar \frac{\partial \psi (x,t)}{\partial t}=-\frac{\hbar^{2}}{2m}\frac{\partial^{2}\psi (x,t)}{\partial x^{2}}+V(x)\psi (x,t).$$

We now proceed by taking higher-order derivatives to remove the imaginary unit $i=\sqrt{-1}$. The time derivative of equation (2.4) in association with equation (2.3) gives

2.8 $$\frac{\partial^{2}\psi (x,t)}{\partial t^{2}}=-\frac{E^{2}}{\hbar^{2}}\psi (x,t);$$

whereas by raising the Hamiltonian given by equation (2.1) to the second power gives

2.9 $$E^{2}=H^{2}(x,p)=\frac{\;p^{4}}{4m^{2}}+\frac{\;p^{2}}{m}V(x)+V^{2}(x).$$

Substitution of the expression for *E*^2^ given by equation (2.9) into equation (2.8) yields

2.10 $$\frac{\partial^{2}\psi (x,t)}{\partial t^{2}}=-\frac{1}{\hbar^{2}}\left(\frac{\;p^{4}}{4m^{2}}+\frac{\;p^{2}}{m}V(x)+V^{2}(x)\right)\psi (x,t).$$

Upon differentiating of equation (2.6) in space two more times,

2.11 $$\frac{\partial^{4}\psi (x,t)}{\partial x^{4}}=\frac{\;p^{4}}{\hbar^{4}}\psi (x,t).$$

The substitution of the quantity *p*^4^*ψ*(*x*, *t*) from equation (2.11) and of the quantity *p*^2^*ψ*(*x*, *t*) from equation (2.6) into equation (2.10) gives

2.12 $$-\hbar^{2}\frac{\partial^{2}\psi (x,t)}{\partial t^{2}}=\frac{\hbar^{4}}{4m^{2}}\;\frac{\partial^{4}\psi (x,t)}{\partial x^{4}}-\frac{\hbar^{2}}{m}V(x)\frac{\partial^{2}\psi (x,t)}{\partial x^{2}}+V^{2}(x)\psi (x,t).$$

Equation (2.12) is the one-dimensional version of the real-valued equation (1.1) originally presented by Schrödinger [1,2] which satisfies the quadratic dispersion relation of matter-waves as dictated by equation (2.2). We coin this time-dependent equation: the ‘flexural-shear beam wave equation’ because of the striking similarities with an approximate beam equation that was proposed by Heidebrecht & Smith [13] to model the dynamics of tall buildings which consist of a strong core-wall that offers flexural resistance acting in parallel with the surrounding framing system of the building that offers shear resistance to lateral loads.

## 3. The time-independent flexural-shear beam equation for matter-waves

The corresponding time-independent equation for standing waves (mode shapes) of equation (2.12) is derived with the standard method of separation of variables where *ψ*(*x*, *t*) = *ψ*(*x*)*f*(*t*). Accordingly,

3.1 $$\frac{\partial^{2}\psi (x,t)}{\partial t^{2}}=\psi (x)\;\frac{d^{2}f(t)}{dt^{2}}$$

and

3.2 $$\frac{\partial^{2}\psi (x,t)}{\partial x^{2}}=\frac{d^{2}\psi (x)}{dx^{2}}\;f(t);\;\frac{\partial^{4}\psi (x,t)}{\partial x^{4}}=\frac{d^{4}\psi (x)}{dx^{4}}\;f(t).$$

Substitution of the expressions for the partial derivatives given by equations (3.1) and (3.2) into equation (2.12) and upon dividing with *ψ*(*x*)*f*(*t*) gives

3.3 $$-m\frac{1}{f(t)}\frac{d^{2}f(t)}{dt^{2}}=\frac{\hbar^{2}}{4m}\frac{1}{\psi (x)}\frac{d^{4}\psi (x)}{dx^{4}}-\frac{V(x)}{\psi (x)}\frac{d^{2}\psi (x)}{dx^{2}}+\frac{m}{\hbar^{2}}V^{2}(x).$$

The left-hand side of equation (3.3) is a function of time alone; whereas, the right-hand side is a function of space alone. In this case,

3.4 $$-m\frac{1}{f(t)}\;\frac{d^{2}f(t)}{dt^{2}}=K,$$

where *K* is a spring constant with units [*M*][*T*]^−2^. Accordingly, equation (3.4) is the equation of motion of a harmonic oscillator with a real-valued solution

3.5 f(t)=Asin⁡ωt+Bcos⁡ωt,

where $\omega =\sqrt{K/m}$ is the natural frequency of the harmonic oscillator. Returning to equation (3.3), its right-hand side is also equal to the spring constant *K* = *mω*^2^:

3.6 $$\frac{\hbar^{2}}{4m}\frac{1}{\psi (x)}\frac{d^{4}\psi (x)}{dx^{4}}-\frac{V(x)}{\psi (x)}\frac{d^{2}\psi (x)}{dx^{2}}+\frac{m}{\hbar^{2}}V^{2}(x)=m\omega^{2}.$$

Multiplication of equation (3.6) with $\hbar^{2}\psi (x)/m$ yields the time-independent flexural-shear beam equation for matter-waves:

3.7 $$\frac{\hbar^{4}}{4m^{2}}\frac{d^{4}\psi (x)}{dx^{4}}-\frac{\hbar^{2}}{m}V(x)\frac{d^{2}\psi (x)}{dx^{2}}+V^{2}(x)\psi (x)=E^{2}\psi (x),$$

where E=ℏω is the quantized energy of the elementary particle. The solution of equation (3.7) yields the eigenvalues and eigenmodes. From the first space derivative of equation (2.3), ∂ψ(x,t)/∂x=(i/ℏ)pψ(x,t), we define the standard momentum operator, $\hat{\;p}=-i\hbar (\partial /\partial x)$. Accordingly, from equation (2.6), the momentum square operator ${\hat{\;p}}^{2}=-\hbar^{2}(\partial^{2}/\partial x^{2})$ and from equation (2.1), the Hamiltonian operator is

3.8 $$\hat{H}=\frac{{\hat{\;p}}^{2}}{2m}+V(x)=-\frac{\hbar^{2}}{2m}\frac{\partial^{2}}{\partial x^{2}}+V(x).$$

From equation (3.8), the Hamiltonian square operator ${\hat{H}}^{2}$ assumes the expression

3.9 $${\hat{H}}^{2}=\frac{h^{4}}{4m^{2}}\frac{\partial^{4}}{\partial x^{4}}-\frac{\hbar^{2}}{m}V(x)\frac{\partial^{2}}{\partial x^{2}}+V^{2}(x).$$

Accordingly, by employing the Hamiltonian square operator ${\hat{H}}^{2}$ defined by equation (3.9), the time-independent flexural-shear beam equation (3.7) can be expressed in the compact form

3.10 $${\hat{H}}^{2}\psi (x)=E^{2}\psi (x).$$

It is the Hamiltonian square operator ${\hat{H}}^{2}$ [28,29] that renders equation (3.10) stiffer than the classical time-independent Schrödinger equation $\hat{H}\psi (x)=E\psi (x)$ that was depleted from its original flexural stiffness [1,2].

## 4. Elementary particle trapped in a finite potential square well with strength *V* > 0

**Figure 1.**
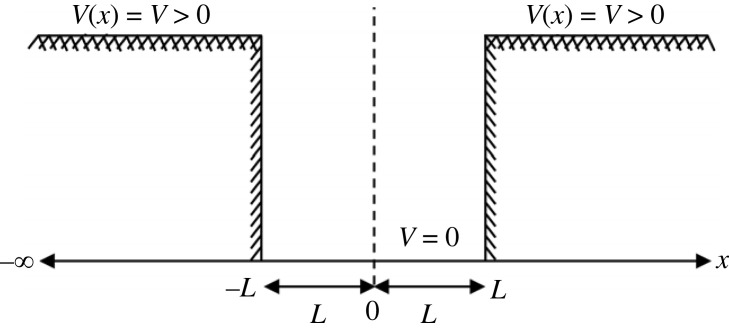
The finite potential square well with constant strength *V* outside the well with width 2*L*.

Given that both the fourth-order, real-valued flexural-shear beam equation (2.12) and the second-order, complex-valued Schrödinger equation (2.7) satisfy the quadratic dispersion relation offered by equation (2.2) as dictated by the Hamiltonian, we proceed by comparing the predictions of these two equations in an effort to show that Schrödinger’s original, fourth-order, real-valued equation (1.1) is a stiffer differential equation than his second-order, complex-valued equation (1.5) or equation (2.7) in one dimension. The quadratic Hamiltonian operator appearing in the flexural-shear beam equation (3.10) leads to elaborate calculations even for simple cases; therefore, we select as a test case the response analysis of an elementary particle with mass *m* trapped in a square potential well with finite potential *V* and width 2*L*. Accordingly, the potential at the bottom of the well is zero as shown in figure 1. This simple, one-dimensional idealization has been employed to determine the wavelengths for colour-centre absorption [30].

For the case where the elementary particle happens to be outside the well (|x|≥L) , *V*(*x*) = *V* > 0 and equation (3.7) gives

4.1 $$\frac{d^{4}\psi (x)}{dx^{4}}-\frac{4m}{\hbar^{2}}V\frac{d^{2}\psi (x)}{dx^{2}}+\frac{4m^{2}}{\hbar^{4}}(V^{2}-E^{2})\psi (x)=0.$$

The solutions of the homogeneous equation (4.1) are expected to be of the form *ψ*(*x*) = e^*βx*^ and equation (3.7) yields the following characteristic equation:

4.2 $$\beta^{4}-\frac{4m}{\hbar^{2}}V\beta^{2}+\frac{4m^{2}}{\hbar^{4}}(V^{2}-E^{2})=0,$$

where *V* > *E* > 0. The four roots of the characteristic equation (4.2) are

4.3 $$\beta_{1}=\frac{1}{\hbar }\sqrt{2m(V+E)}>0,\;\beta_{2}=-\frac{1}{\hbar }\sqrt{2m(V+E)}=-\beta_{1}$$

and

4.4 $$\beta_{3}=\frac{1}{\hbar }\sqrt{2m(V-E)}>0,\;\beta_{4}=-\frac{1}{\hbar }\sqrt{2m(V-E)}=-\beta_{3}.$$

Accordingly, for the case |*x*| ≥ *L*, where *V*(*x*) = *V* > *E* > 0, the solution for *ψ*(*x*) is

4.5 $$\psi (x)=A_{1}\;e^{\beta_{1}x}+A_{2}\;e^{-\beta_{1}x}+A_{3}\;e^{\beta_{3}x}+A_{4}\;e^{-\beta_{3}x}.$$

For the case where the elementary particle is within the potential well (|*x*| ≤ *L*), *V*(*x*) = 0 and equation (3.7) gives

4.6 $$\frac{d^{4}\psi (x)}{dx^{4}}-\frac{4m^{2}}{\hbar^{4}}E^{2}\psi (x)=0.$$

By setting ${(4m^{2}/\hbar^{4})E}^{2}=k^{4}$, equation (4.6) assumes the form

4.7 $$\frac{d^{4}\psi (x)}{dx^{4}}-k^{4}\psi (x)=0.\;$$

Equation (4.7) has a real-valued solution [31,32]:

4.8 $$\psi (x)=C_{1}\sin\left(kx\right)+C_{2}\cos\left(kx\right)+C_{3}\sinh\left(kx\right)+C_{4}\cosh\left(kx\right),$$

where $k=(1/\hbar )\sqrt{2mE}$ is a positive, real wavenumber. In this case (*x* ≤ |*L*|), *V*(*x*) = 0 and from equation (2.1), *E* = *p*^2^/(2*m*); therefore, the wavenumber $k=(1/\hbar )\sqrt{2mE}$ appearing in equation (4.8) is $k=(1/\hbar )\sqrt{2mp^{2}/(2m)}=p/\hbar$ which is the de Broglie wavenumber. This supports the choice for the same symbol, *k*.

It is worth noting that equation (4.7) is the equation of motion of a vibrating flexural beam with distributed mass per length $\bar{m}$ with units [*M*][*L*]^−1^, Young’s modulus of elasticity *Y* with units [*M*][*L*]^−1^[*T*]^−2^ (*force*/*area*) and moment of cross-sectional area *I* with units [*L*]^4^. For a vibrating flexural beam $k^{4}=\bar{m}\omega^{2}/YI$ and upon using E=ℏω and cancelling the angular frequency *ω*, we obtain the analogy $YI/\bar{m}⟶{\left(\hbar /2m\right)}^{2}$, both having units of [*L*]^4^[*T*]^−2^.

### 4.1. Continuity of solutions

#### 4.1.1. Case 1: *x* ≤ −*L* where *V*(*x*) = *V* and *V* − *E* > 0. Bound states

For this case where *x* ≤ −*L*, the solution *ψ*(*x*) given by equation (4.5) remains finite when *A*_2_ = *A*_4_ = 0. Consequently, for this case

4.9 $$\psi (x)=A_{1}\;e^{\beta_{1}x}+A_{3}\;e^{\beta_{3}x}\;\mathrm{for}\;x\leq -L,$$

in which *β*_1_ and *β*_3_ are real-valued and given by equations (4.3) and (4.4).

#### 4.1.2. Case 2: −*L* ≤ *x* ≤ *L* where *V*(*x*) = 0

For this case *ψ*(*x*) is given by equation (4.8).

#### 4.1.3. Case 3: *x* ≥ *L* where *V*(*x*) = *V* and *V* − *E* > 0. Bound states

For this case where *x* > *L*, the solution *ψ*(*x*) given by equation (4.5) remains finite when *A*_1_ = *A*_3_ = 0. Consequently, for this case

4.10 $$\psi (x)=A_{2}\;e^{-\beta_{1}x}+A_{4}\;e^{-\beta_{3}x}\;\mathrm{for}\;x\geq L,$$

in which *β*_1_ and *β*_3_ are real-valued and given by equations (4.3) and (4.4).

The solution of the wave equation *ψ*(*x*) has to be continuous over the entire domain −∞ < *x* < ∞. Accordingly, at *x* = −*L*, equation (4.9) from the left and equation (4.8) from the right need to satisfy the following continuity equations:

4.11*a* $$\psi (-L^{-})=\psi (-L^{+}),\;\frac{d\psi (-L^{-})}{dx}=\frac{d\psi (-L^{+})}{dx}$$

and

4.11*b* $$\frac{d^{2}\psi (-L^{-})}{dx^{2}}=\frac{d^{2}\psi (-L^{+})}{d^{2}x},\;\frac{d^{3}\psi (-L^{-})}{d^{3}x}=\frac{d^{3}\psi (-L^{+})}{d^{3}x}.$$

Similarly, at *x* = *L*, equation (4.8) from the left and equation (4.10) from the right need to satisfy the following continuity equations:

4.12*a* $$\psi (L^{-})=\psi (L^{+}),\;\frac{d\psi (L^{-})}{dx}=\frac{d\psi (L^{+})}{dx}$$

and

4.12*b* $$\frac{d^{2}\psi (L^{-})}{dx^{2}}=\frac{d^{2}\psi (L^{+})}{d^{2}x},\;\frac{d^{3}\psi (L^{-})}{d^{3}x}=\frac{d^{3}\psi (L^{+})}{d^{3}x}.$$

The eight continuity equations given by equations (4.11) and (4.12) form a homogeneous system of eight equations which yields the eigenvalues *z*_*n*_ = *k*_*n*_*L* and eigenfunctions (mode shapes) *ψ*_*n*_(*x*) of the wave function *ψ*(*x*).

### 4.2. Eigenvalue analysis

The wavenumbers *β*_1_ and *β*_3_ given by equation (4.3) and (4.4) can be expressed as

4.13 $$\beta_{1}=\sqrt{\frac{2mV}{\hbar^{2}}+\frac{2mE}{\hbar^{2}}}=\sqrt{b^{2}+k^{2}}$$

and

4.14 $$\beta_{3}=\sqrt{\frac{2mV}{\hbar^{2}}-\frac{2mE}{\hbar^{2}}}=\sqrt{b^{2}-k^{2}},$$

where $b=(1/\hbar )\sqrt{2mV}$ is a positive number and $k=(1/\hbar )\sqrt{2mE}=2\pi /\lambda =p/\hbar$ is the wavenumber of the solution of *ψ*(*x*) when −*L* ≤ *x* ≤ *L* given by equation (4.8).

The homogeneous system of eight equations that is generated by the eight continuity equations (4.11) and (4.12) can be decomposed into four equations that produce the even eigenfunctions $\psi_{n}^{e}(x)$ and four equations that produce the odd eigenfunctions $\psi_{n}^{o}(x)$. The homogeneous system that produces the even eigenfunctions is

4.15 $$\left[\begin{array}{cccc} \cos\left(z\right) & \cosh\left(z\right) & \;-e^{-\sqrt{b^{2}L^{2}+z^{2}}} & -e^{-\sqrt{b^{2}L^{2}-z^{2}}}\\ -z\sin\left(z\right) & z\sinh\left(z\right) & \sqrt{b^{2}L^{2}+z^{2}}\;e^{-\sqrt{b^{2}L^{2}+z^{2}}}\; & \sqrt{b^{2}L^{2}-z^{2}}\;e^{-\sqrt{b^{2}L^{2}-z^{2}}}\\ -z^{2}\cos\left(z\right) & z^{2}\cosh\left(z\right) & -(b^{2}L^{2}+z^{2})\;e^{-\sqrt{b^{2}L^{2}+z^{2}}}\; & -(b^{2}L^{2}-z^{2})\;e^{-\sqrt{b^{2}L^{2}-z^{2}}}\\ z^{3}\sin\left(z\right) & z^{3}\sinh\left(z\right) & {\left(b^{2}L^{2}+z^{2}\right)}^{3/2}\;e^{-\sqrt{b^{2}L^{2}+z^{2}}} & {\left(b^{2}L^{2}-z^{2}\right)}^{3/2}\;e^{-\sqrt{b^{2}L^{2}-z^{2}}} \end{array}\right]\left\{\begin{array}{c} C2\\ C4\\ A2\\ A4 \end{array}\right\}=0,$$

where $bL=(L/\hbar )\sqrt{2mV}$ is a dimensionless positive real number that expresses the strength of the potential well and $z=kL=(L/\hbar )\sqrt{2mE}$ are the eigenvalues of the even eigenfunctions to be determined. The eigenvalues *z*_*n*_ depend on the dimensionless product *bL* rather than on the individual values of *b* and *L* and they are calculated by setting the determinant of the 4 × 4 matrix appearing on the left of equation (4.15) equal to zero. As an example, for *bL* = 10 the characteristic equation of the homogeneous system given by equation (4.15) yields four real roots (eigenvalues, *n* ∈ {1, 3, 5, 7}) for $z_{n}=(L/\hbar )\sqrt{2mE_{n}}=1.9747,\;4.6204,\;7.2901$ and 9.7999. For larger values of *bL* (deeper and wider potential well) the number of real eigenvalues increases given that the unknown eigenvalue *z* needs to remain smaller than *bL* for the radical $\sqrt{b^{2}L^{2}-z^{2}}$ of the last column of the matrix appearing in equation (4.15) to remain positive.

Similarly, the homogeneous system as results from the continuity equations that produces the odd eigenfunctions is

4.16 [sin⁡(z)sinh⁡(z)−e−b2L2+z2−e−b2L2−z2    zcos⁡(z)zcosh⁡(z)b2L2+z2 e−b2L2+z2 b2L2−z2 e−b2L2−z2    −z2sin⁡(z)z2sinh⁡(z)−(b2L2+z2) e−b2L2+z2 −(b2L2−z2) e−b2L2−z2    −z3cos⁡(z)z3cosh⁡(z)(b2L2+z2)3/2 e−b2L2+z2(b2L2−z2)3/2 e−b2L2−z2]{C1C3A2A4}=0.

The finite eigenvalues $z_{n}=(L/\hbar )\sqrt{2mE_{n}}$ that correspond to the odd eigenfunctions are computed by setting the determinant of the 4 × 4 matrix appearing on the left of equation (4.16) equal to zero. As an example, for *bL* = 10 the characteristic equation of the homogeneous system given by equation (4.16) yields three real roots (eigenvalues, *n* ∈ {2, 4, 6}) for $z_{n}=(L/\hbar )\sqrt{2mE_{n}}=3.2887,\;5.9574$ and 8.5976. For larger values of *bL* (deeper and wider potential well) the number of real roots of the characteristic equation (eigenvalues) increases as long as *z* < *bL* so that the radical $\sqrt{b^{2}L^{2}-z^{2}}$ appearing in the last column of the 4 × 4 matrix equation (4.16) remains real.

## 5. Comparison of the eigenvalues predicted from the fourth-order flexural-shear beam equation and from the classical second-order Schrödinger equation

For any given value of the strength of the square potential well, *bL*, the resulting eigenvalues of the fourth-order, flexural-shear beam equation (3.7) or (3.10), $z_{n}=(L/\hbar )\sqrt{2mE_{n}}$, yield the admissible energy levels of the elementary particle in the finite square potential well, $E_{n}=(z_{n}^{2}\;\hbar^{2})/(2mL^{2})$. Clearly, the predicted energy levels, *E*_*n*_, are different from the corresponding energy levels, *E*_*n*_, predicted from the solution of the second-order, time-independent Schrödinger equation.

The predicted eigenvalues $z_{n}=(L/\hbar )\sqrt{2mE_{n}}$ of an elementary particle in a finite square potential well with the second-order, Schrödinger equation are the roots of the transcendental equations (5.1) and (5.2) [33]:

5.1 $$\tan\left(z\right)=\sqrt{\frac{b^{2}L^{2}}{z^{2}}-1}\;\text{for even eigenfunctions}$$

and

5.2 $$\cot\left(z\right)=-\sqrt{\frac{b^{2}L^{2}}{z^{2}}-1}\;\text{for odd eigenfunctions},$$

where $b=(1/\hbar )\sqrt{2mV}$ as in the previous analysis.

As an example for *bL* = 10, equation (5.1) yields four real roots (eigenvalues of the even eigenfunctions, *n* ∈ {1, 3, 5, 7}) for $z_{n}=(L/\hbar )\sqrt{2mE_{n}}=1.\;4276,\;4.2711,\;7.0689,\;9.6789$; and equation (5.2) yields three real roots (eigenvalues of the odd eigenfunctions, *n* ∈ {2, 4, 6}) for $z_{n}=(L/\hbar )\sqrt{2mE_{n}}=2.8523,\;5.6792$ and 8.4232.

Table 1 compares the predicted eigenvalues for a non-relativistic particle in a finite square potential well with potential *V* from the fourth-order, flexural-shear beam wave equation and the second-order, Schrödinger wave equation for bL=10 and 30. Table 1 also shows the limiting eigenvalues for a particle trapped in an infinitely deep potential well (*V* = ∞) as they result from the second-order, Schrödinger equation, $z_{n}=(L/\hbar )\sqrt{2mE_{n}}=n\pi /2$ [33], and from the fourth-order, flexural-shear beam equation which are the solutions of the characteristic equation cos⁡(2kL)cosh⁡(2kL)=1 as shown in the following.

**Table 1.** The seven eigenvalues (energy levels) $z_{n}=(L/\hbar )\sqrt{2mE_{n}}$ for a particle in a finite potential well with strength $bL=(L/\hbar )\sqrt{2mV}=10$, when described with the fourth-order, flexural-shear beam wave equation and with the classical second-order, Schrödinger wave equation, together with the first nine corresponding eigenvalues when bL=30 and ∞.

	fourth-order flexural-shear beam equation			second-order Schrödinger equation		
	$bL=(L/\hbar )\sqrt{2mV}$		cos⁡(2z)=1/cosh⁡(2z)	$bL=(L/\hbar )\sqrt{2mV}$		$z_{n}=(L/\hbar )\sqrt{2mE_{n}}=n\pi /2$
no. eigenvalue $z_{n}=(L/\hbar )\sqrt{2mE_{n}}$	*bL* = 10	*bL* = 30	*bL* = ∞	*bL* = 10	*bL* = 30	*bL* = ∞
*n* = 1	1.974707	2.217448	2.365020	1.427552	1.520104	*π*/2 = 1.570796
*n* = 2	3.288725	3.682318	3.926602	2.852342	3.040082	*π* = 3.141593
*n* = 3	4.620365	5.157210	5.497804	4.271095	4.559804	3*π*/2 = 4.712389
*n* = 4	5.957359	6.633016	7.068583	5.679208	6.079134	2*π* = 6.283185
*n* = 5	7.290139	8.113046	8.639380	7.068891	7.597928	5*π*/2 = 7.853982
*n* = 6	8.597635	9.589274	10.210176	8.423204	9.116028	3*π* = 9.424778
*n* = 7	9.799891	11.06978	11.780972	9.678884	10.633257	7*π*/2 = 10.995574
*n* = 8	…	12.55174	13.351769	…	12.149413	4*π* = 12.566371
*n* = 9	…	14.03491	14.922565	…	13.664261	9*π*/2 = 14.137167
⋮	…	⋮	⋮	…	⋮	⋮

Table 1 reveals that when *bL* = 10 all seven eigenvalues that result from the fourth-order, flexural-shear beam equation are larger than the corresponding seven eigenvalues that result from the classical second-order, Schrödinger equation. The same is true for the case when *bL* = 30. Consequently, this analysis shows that the fourth-order, real-valued flexural-shear beam equation for matter-waves given by equation (2.12) is a stiffer equation than the classical second-order, complex-valued Schrödinger equation given by equation (2.7). Therefore, Schrödinger’s equivalence statement that equation (1.5) (which is eqn (4″) in his 1926 paper [1]) and equation (1.1) (which is eqn (4) in his 1926 paper [1]) are equivalent, is not true.

Furthermore, table 1 reveals that when *bL* = 10, the first two eigenvalues *z*_1_ = 1.9747 and *z*_2_ = 3.2887 that result from the fourth-order, flexural-shear beam equation are even larger than the first two eigenvalues *z*_1_ = *π*/2 and *z*_2_ = *π* that result from the classical second-order, Schrödinger equation at the limiting case when the strength of the potential well is infinite ($bL=(L/\hbar )\sqrt{2mV}=\infty$) [33]. This pattern where the eigenvalues predicted from the fourth-order, flexural-shear beam equation when trapped in a finite potential well exceed the eigenvalues predicted by the second-order, Schrödinger equation when the particle is trapped in an infinite potential well becomes more dominant as the strength *bL* of the finite potential well increases. For instance, when *bL* = 30, the first seven eigenvalues that result from the fourth-order, flexural-shear beam equation are larger than the first seven eigenvalues that result from the classical second-order, Schrödinger equation at the limiting case of an infinitely strong potential well. Accordingly, there is a need to calculate the energy levels of an elementary particle trapped in an infinitely strong potential well (*bL* = ∞) when described with the fourth-order, flexural-shear beam wave equation (3.7) or (3.10).

The wave functions (eigenmodes) associated with the energy levels (eigenvalues) appearing in table 1 for the situation where the elementary particle is described with the fourth-order, flexural-shear beam wave function are offered by equation (4.8) for 0 ≤ |*x*| ≤ *L* and by equation (4.10) for *x* ≥ *L*. Accordingly, the even eigenfunctions (*n* ∈ {1, 3, 5, …}) are given by

5.3 $$\psi_{n}^{e}(x)=C_{2}\cos\left(z_{n}\frac{x}{L}\right)+C_{4}\cosh\left(z_{n}\frac{x}{L}\right)\;\text{for}\;0\leq |x|<L$$

and

5.4 $$\psi_{n}^{e}(x)=A_{2}\;e^{-(x/L)\sqrt{b^{2}L^{2}+z_{n}^{2}}}+A_{4}\;e^{-(x/L)\sqrt{b^{2}L^{2}-z_{n}^{2}}}\;\mathrm{for}\;L<x,$$

whereas, the odd eigenfunctions (*n* ∈ {2, 4, 6, …}) are given by

5.5 $$\psi_{n}^{o}(x)=C_{1}\sin\left(z_{n}\frac{x}{L}\right)+C_{3}\sinh\left(z_{n}\frac{x}{L}\right)\;\mathrm{for}\;0\leq |x|<L,$$

and $\psi_{n}^{o}(x)$ is given again by equation (5.4) for *L* < *x*.

The coefficients *C*_2_, *C*_4_, *A*_2_ and *A*_4_ appearing in equations (5.3) and (5.4) are obtained upon solving the homogeneous system of equations given by the matrix equation (4.15); whereas, the coefficients *C*_1_, *C*_2_, *A*_2_ and *A*_4_ appearing in equations (5.5) and (6.1*a*) are obtained upon solving the homogeneous system of equations given by the matrix equation (4.16). When solving the homogeneous system of equations, one of the four coefficients is assigned an arbitrary value and the other three coefficients are calculated in proportion to the arbitrary assigned value of the first coefficient since the eigenfunctions $\psi_{n}^{e}(x)$ and $\psi_{n}^{o}(x)$ are eigenmodes of arbitrary amplitude which subsequently can be normalized according to some normalization rule such as $\int_{-\infty }^{\infty }|\psi (x){|}^{2}\;dx=\int_{-\infty }^{\infty }\psi^{2}(x)\;dx=1$.

**Figure 2.**
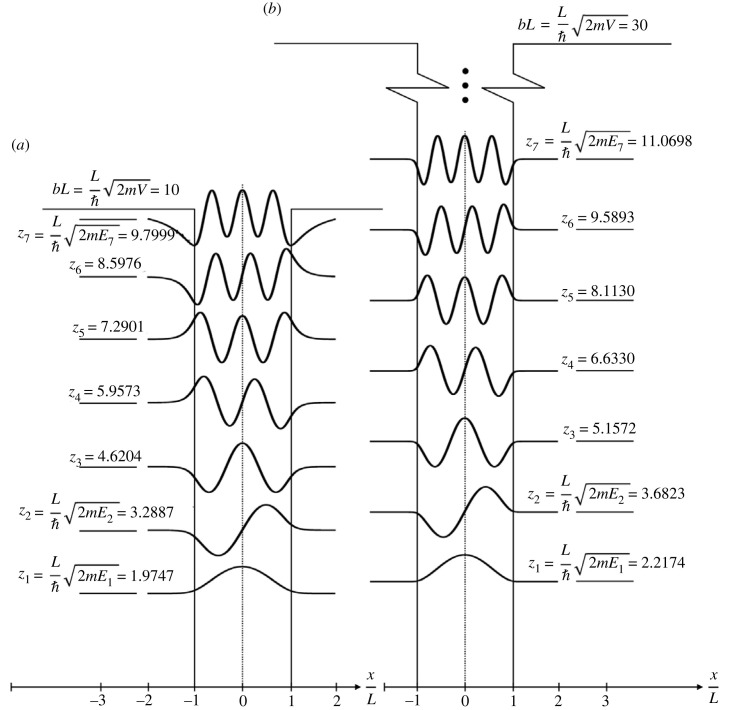
The seven eigenfunctions *ψ*_*n*_(*x*), *n* ∈ {1, 2, …, 7}, of an elementary particle described with the fourth-order, flexural-shear beam equation (3.7) or (3.10) when trapped in a potential well with finite strength $bL=(L/\hbar )\sqrt{2mV}=10$ which manifest at the energy levels $E_{n}=z_{n}^{2}\hbar^{2}/2mL^{2}$ (*a*); together with the corresponding first seven eigenfunctions *ψ*_*n*_(*x*) when the elementary particle is trapped in a potential well with finite strength $bL=(L/\hbar )\sqrt{2mV}=30$ (*b*).

Figure 2*a* plots the seven eigenfunctions *ψ*_*n*_(*x*), *n* ∈ {1, 2, …, 7}, of an elementary, non-relativistic particle described with the fourth-order, flexural-shear beam equation (3.7) or (3.10) when trapped in a potential well with finite strength, $bL=(L/\hbar )\sqrt{2mV}=10$, which manifest at the energy levels $E_{n}=z_{n}^{2}\hbar^{2}/2mL^{2}$. The eigenvalues *z*_*n*_ are listed in table 1. Figure 2*b* plots the corresponding first seven wave functions *ψ*_*n*_(*x*) (there are 19 wave functions in total) when the elementary particle is trapped in a potential well with finite strength *bL* = 30.

## 6. Eigenvalues of the fourth-order matter-wave equation of an elementary particle trapped in an infinite-potential square well

Figure 2 reveals that as the strength of the finite potential well increases, the eigenfunctions *ψ*_*n*_(*x*) that result from the solution of the fourth-order wave equation (3.7) or (3.10) meet the walls of the square potential well at a decreasing slope which eventually tends to zero, d*ψ*/d*x* (*x* = −*L*) = (d*ψ*/d*x*)(*x* = *L*) = 0, as the strength of the potential well, *bL*, tends to infinity.

These zero-slope boundary conditions of the eigenmodes of the trapped particle at the walls of the infinitely strong potential well are drastically different from the finite-slope boundary conditions of the eigenmodes of the trapped particle when described with the second-order Schrödinger equation ($\psi_{n}(x)=\sqrt{2/a}\sin\left((n\pi /a)x\right)\;\mathrm{with}\;0<x\leq a=2L$) [33]. These fixed-end (zero-slope) boundary conditions (clamped eigenmodes) are another proof that the fourth-order, real-valued equation (1.1) originally proposed by Schrödinger [1,2] is a stiffer equation than his classical second-order, complex-valued equation (1.5).

The eigenfunctions of the particle trapped in an infinitely strong potential well when described with the fourth-order, flexural-shear beam wave equation (3.7) are given by equation (4.8), and the integration constants *C*_1_, *C*_2_, *C*_3_ and *C*_4_ are derived by enforcing the boundary conditions

6.1*a* ψ(−L)=ψ(L)=0

and

6.1*b* $$\frac{d\psi (-L)}{dx}=\frac{d\psi (L)}{dx}=0.$$

This homogeneous system of four equations results in the transcendental characteristic equation

6.2 cos⁡(2kL)cosh⁡(2kL)=1.

The roots of equation (6.2), $z_{n}=k_{n}L=(L/\hbar )\sqrt{2mE_{n}}$, are the eigenvalues of the fixed-end eigenmodes appearing in table 1 under *bL* = ∞.

## 7. Conclusion

In this paper, we show that Schrödinger’s original fourth-order, real-valued equation (1.1) for matter-waves is a stiffer description (higher energy levels) of the behaviour of elementary particles than the description offered from his classical, second-order, complex-valued equation (1.5). Given the remarkable predictions of the complex-valued equation (1.5) for the energy levels of the chemical elements as manifested from their observed atomic line spectra together with the features of the Periodic Table [15–19,25], in association with that his original fourth-order, real-valued equation predicts invariably higher energy levels (therefore, apparently incorrect), this paper shows that quantum mechanics can only be described with the less stiff, complex-valued wave equation (1.5). This finding is in agreement with more elaborate recent studies [20–24].

## Data Availability

This work did not require ethical approval from a human subject or animal welfare committee.

## Data accessibility

This article does not contain any additional data.
